# Aquatic and Terrestrial Invertebrate Welfare

**DOI:** 10.3390/ani13213375

**Published:** 2023-10-31

**Authors:** Gregory A. Lewbart, Trevor T. Zachariah

**Affiliations:** 1 College of Veterinary Medicine, NC State University, Raleigh, NC 27607, USA; 2 Brevard Zoo|Sea Turtle Healing Center, 8225 North Wickham Road, Melbourne, FL 32940, USA; tzachariah@brevardzoo.org

**Keywords:** invertebrates, welfare, enrichment, health, coelenterates, chelicerates, insects, echinoderms, mollusks, crustaceans, myriapods

## Abstract

**Simple Summary:**

Invertebrates (animals without backbones) make up over 95% of the earth’s species yet compared with vertebrates (animals with backbones like fishes, amphibians, reptiles, birds, and mammals) our understanding of and efforts relating to the topic of welfare is relatively minimal. We have selected seven of the most economically important and widely recognized invertebrate taxa to focus the topic of animal welfare on. In these pages the reader will learn about coelenterates (jellyfishes, anemones, and corals), mollusks (snails, slugs, squid, and octopi), crustaceans (lobsters, crabs, and shrimp), echinoderms (sea stars, sea urchins, and sea cucumbers), chelicerates (spiders, scorpions, and horseshoe crabs), myriapods (centipedes and millipedes), and insects (butterflies, honeybees, and fruit flies). In addition to discussing the welfare of these species, other topics, including anatomy, physiology, husbandry, natural history, and environmental diseases, are reviewed.

**Abstract:**

Invertebrates are a diverse group of animals that make up the majority of the animal kingdom and encompass a wide array of species with varying adaptations and characteristics. Invertebrates are found in nearly all of the world’s habitats, including aquatic, marine, and terrestrial environments. There are many misconceptions about invertebrate sentience, welfare requirements, the need for environmental enrichment, and overall care and husbandry for this amazing group of animals. This review addresses these topics and more for a select group of invertebrates with biomedical, economical, display, and human companionship importance.

## 1. Introduction

Before we arrive at the topic of invertebrate welfare we should first answer the question, “what are invertebrates?” Invertebrates are a diverse group of animals that lack a vertebral column or backbone. They make up the majority of the animal kingdom and encompass a wide array of species (over 95% of those described) with varying adaptations and characteristics. Invertebrates are found in nearly all of the world’s habitats, including aquatic, marine, and terrestrial environments.

Some fundamental characteristics of invertebrates:

1. Absence of a backbone: The most defining characteristic of invertebrates is the absence of a vertebral column (backbone or spine). This distinguishes them from vertebrates, which are animals with a well-developed internal skeleton composed of cartilaginous or bony vertebrae.
2. Diverse body structures: Invertebrates exhibit an incredible diversity of body forms and structures. They include animals with soft bodies, hard exoskeletons, and a variety of appendages for feeding, locomotion, reproduction, defense, and other functions.
3. Exoskeletons: Many invertebrate groups have exoskeletons, which are external hard coverings usually made of chitin. These exoskeletons provide protection, support, and a firm surface for muscle attachment. In some cases, these exoskeletons must be shed (a process called molting) to accommodate growth and heal injuries or defects.
4. Nervous System: Invertebrates have a wide range of nervous system complexities, from simple nerve nets (jellyfish) to more complex systems with well-defined brains and sensory organs (some mollusks, arachnids, crustaceans, and insects).
5. Reproduction: Invertebrates employ various reproductive strategies, including sexual reproduction (with internal or external fertilization) and asexual reproduction (such as budding or fragmentation).
6. Segmentation: Some invertebrates exhibit segmentation, where their bodies are divided into repeating segments. Each segment may have specialized structures, such as gills, legs, or sensory organs. Examples include the annelids, crustaceans, and insects.
7. Habitats: Invertebrates inhabit virtually every ecosystem on earth, from the highest mountainous regions to deep-sea hydrothermal vents and virtually everything in between. They play crucial roles as decomposers, pollinators, predators, and as prey.
8. Taxonomic Diversity: Invertebrates encompass a wide variety of taxonomic groups, including coelenterates (jellyfish, anemones, corals), mollusks (snails, clams, octopuses), annelids (oligochaetes, polychaetes, leeches), arthropods (insects, spiders, crustaceans, arachnids), echinoderms (sea stars, sea urchins, sea cucumbers), and many more.

Due to their incredible diversity and ecological importance, invertebrates are the focus of extensive scientific research and conservation efforts. Invertebrates contribute to ecosystem functioning and provide valuable insights into evolutionary processes and adaptation.

There are currently over 40 recognized phyla of invertebrates (not including the protozoans). Many of these phyla are little known to veterinary medicine, but for no better reason than they contain few species, have microscopic representatives, or possess no obvious economic value. Each phylum and its members are important to the diversity and survival of the planet, even if the group is only studied by a relatively small number of scientists. Writing a comprehensive article for the welfare of all invertebrate groups would be an inefficient task. Thus, we have elected to include information on the metazoan taxa frequently encountered by veterinarians and researchers.

The elevated awareness and influence of animal welfare and changing public opinion have led to increased consideration for the welfare of invertebrates. For researchers, the efforts to reduce, refine, and replace the use of vertebrates have resulted in the increased use and focus on invertebrate animal models. The perception that invertebrates are more acceptable than vertebrates for research purposes is largely due to the belief that invertebrates do not experience pain. There are no regulations or restrictions in the United States on invertebrate use in research. Despite growing concerns from various individuals, institutions, and organizations, the Animal Welfare Act and Public Health Service Policy do not protect invertebrates. Some organizations, including many aquariums and zoos, require a protocol for invertebrates to be used in research. The United States Department of Agriculture (USDA) has an “Information Resource on the Care and Use of Invertebrates” (AWIC Resource Series No. 8) that contains references for research using invertebrates. The Association of Zoos and Aquariums (AZA) has published care manuals for giant Pacific octopus (GPO), Japanese spider crab, and jellyfish. The USDA has standards for non-indigenous arthropods (USDA APHIS, 2002) but these guidelines are meant to prevent the release of invasive species. The 8th edition of the UFAW Handbook on the Care and Management of Laboratory Animals contains a section on cephalopods with references [1]. Several reviews, references and recommendations have been published on behavior and husbandry of invertebrate species [2,3,4,5,6].

The discussion about invertebrate pain recognition is gaining traction and changes have been initiated by the United Kingdom and other European countries [7,8,9,10]. As veterinary interest in invertebrate medicine increases, more research and scientific findings leading to appropriate care for invertebrates in clinical and laboratory settings will follow. It is possible that some invertebrates will soon be included in U.S. animal welfare regulations leading to regulatory oversight. Cephalopods are protected in the European Union under EU Directive 2010/63/EU and in Australia, Canada, and the United Kingdom. Decapod crustaceans and cephalopods are protected in New Zealand, Norway, and Switzerland [5]. Sentience in some cephalopods and decapods was reviewed with implications for animal welfare regulations [11,12]. Criteria evaluated included (1) possession of nociceptors, (2) connections between nociceptors and integrative brain regions, (3) associative learning beyond habituation and sensitization, (4) flexible self-protective behaviors in response to injury and threat, (5) behavior demonstrating the animal benefits from local anesthetics or analgesics when injured, (6) motivational trade-offs demonstrating balancing of threat versus reward, (7) possession of integrative brain regions, (8) associative learning beyond habituation and sensitization. Based on these criteria, Birch [13] concluded there is very strong evidence of sentience in octopods and strong evidence for sentience in true crabs (infraorder Brachyura).

## 2. Coelenterates

### 2.1. Taxonomy

This large group includes the comb jellies (Ctenophores), Hydrozoans (hydras, fire coral, Portuguese Man-O-War), Scyphozoans (jellyfishes), and Anthozoans (soft corals, stony corals, and sea anemones). The Anthozoa are the most evolutionarily advanced group of coelenterates. While both soft (gorgonians) and hard (stony) corals are colonial, anemones are typically solitary [14,15].

### 2.2. Natural History

▪ This is an economically important assembly of animals for environmental monitoring, private and public display, tourism [16], and research. Jellyfish exhibits are popular displays in public aquariums, upscale restaurants, corporate offices, and even private homes in affluent communities. Coral reefs are probably the most beautiful, fragile, and diverse ecosystems on earth. Research on coral diseases and demise are one of the most active areas of marine research.
▪ Coelenterates lack a brain (distinct central neural ganglion) and rely on a “nerve net” for motor and sensory transmissions. The coelenterate nerve net was described in detail almost 90 years ago in three important papers [17,18,19,20]. In the 1950′s slow motion videography proved that anemones contract, sway, and display other movements without stimulation [17,21].
▪ It is beyond the scope of this article to include details of anatomy, physiology, and taxonomy, but the basics are included here.

### 2.3. Environment

**Figure 1:**
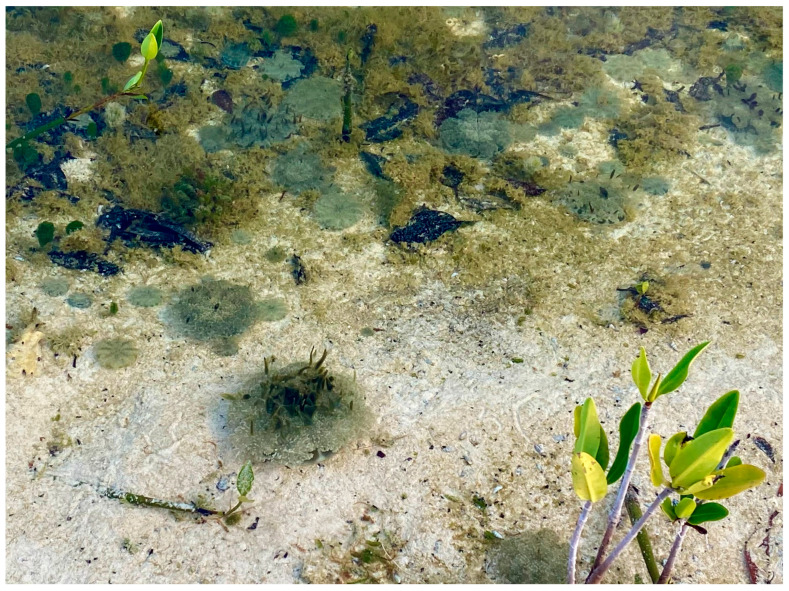
A population of upside-down jellyfish (*Cassiopea* sp.) in the Turks and Caicos Islands. These unusual jellyfish possess symbiotic zooxanthellae in their tissues to assist with nutrition, much like their cousins the corals. Photograph by GA Lewbart.

Most coelenterates are marine and there are individual and colonial forms. One of the most interesting and extensively studied jellies is the upside-down jellyfish, *Cassiopeia* spp. [22,23] (Figure 1). These tropical and sub-tropical marine coelenterates orient upside-down with the dorsal surface of the bell on the bottom and the manubria and tentacles reaching towards the water’s surface [23]. Like most corals and some anemones these jellies host photosynthetic dinoflagellates, which provide the jellyfish with supplemental nourishment. The upside-down jellyfish’s unique natural history make them important for study in a number of scientific areas.

### 2.4. Nutrition

Coelenterates possess a single gastrointestinal tract opening and most have stinging cells termed cnidocytes, with contained nematocysts, that are utilized for defense and prey capture. These cnidocytes are located on the tentacles of corals and anemones. The Caribbean and Gulf of Mexico coral reef scleractinian coral *Mycetophyllia reesi* [24], is an exception. This coral does not possess tentacles and uses mucus strands to entangle prey prior to ingestion [24]. Nearly all corals, and some jellies (e.g., *Cassiopea* sp.; Figure 1), obtain supplemental and in some cases necessary nutrition from symbiotic organisms living with their soft tissues [14].

### 2.5. Physical Health

**Figure 2:**
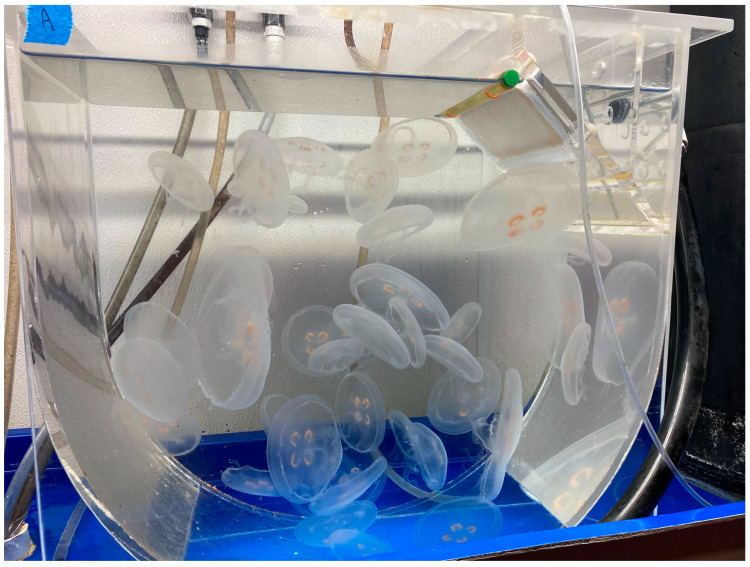
For jellies to thrive under human care they require certain water flow and other life support system parameters. The kreisel, pictured here, is harboring a population of captive-reared moon jellies (*Aurelia aurita*). This type of system is critical to the health and welfare of pelagic coelenterates. Photograph by GA Lewbart.

In recent years, numerous published studies have examined the effect of anthropogenic activities on corals, including climate change and coral diseases in general [25,26,27,28,29,30,31,32,33]. While a complete health review is beyond the scope of this article, it is important to understand the global importance of corals and the many efforts underway to protect them from a veterinary perspective. Coelenterates under human care require very specific life support systems (e.g., reef aquaria and jellyfish displays). For pelagic jellies to thrive they require a constant, circular, slow current that is produced in cylindrical systems called kreisels (the German word for gyro) (Figure 2). For a summary of coelenterate diseases see Stoskopf et al. [34].

### 2.6. Behavior

“What’s on the mind of a jellyfish? A review of behavioral observations on *Aurelia* sp. jellyfish” is a fascinating article about moon jellyfish (*Aurelia* sp.) behavior [35]. The author describes that jellyfish do not simply drift about the oceans subject to currents, tides, and wind. In fact, jellyfish have an elaborate sensory system rich with chemoreceptors, gravity sensors, hydrostatic pressure receptors, nerves, photoreceptors, pressure sensors, and the ability to sense direction [35,36,37]. At least 10 different behaviors have been attributed to *Aurelia* sp. [35]. Jellies, at least *Aurelia aurita* (the moon jelly), can use chemoreception (taste) to discriminate between agar pellets containing edible brine shrimp (*Artemia* sp.) and those without. The non-food agar pellets were secured by the tentacles and discarded into the currents produced by bell pulsations while edible pellets were transferred via ciliary movement to the oral cavity [38,39].

### 2.7. Mental/Psychological Health and Welfare

Briffa and Greenaway [40] is one of the few examples in the literature where a “personality” has been assigned to an invertebrate. The authors define “animal personality” as the way individuals differ from one another in either single behaviours or suites of related behaviours in a way that is consistent over time [40]. They noted that individual anemones (*Actinia equina*) had their own behavior patterns and startle responses. 

Coelenterates are exploited for the pet trade, public display, research, tourism, and even as food. All of these activities and industries negatively impact the welfare of these species. In addition, climate change and global warming are having significant, and mostly negative effects, on coelenterate welfare. As humans we need to keep animal welfare in the forefront of our interactions and impact on these animals. For a thorough review of coelenterate welfare see Weil et al. [41].

## 3. Mollusks

### 3.1. Taxonomy

There are six classes of mollusks. The gastropods (slugs and snails), cephalopods (squid, cuttlefish, octopuses), bivalves (clams, mussels, oysters, scallops), scaphopods (tusk shells), monoplacophora, and polyplacophora (chitons). In this article we will focus on the gastropods and cephalopods.

**Figure 3:**
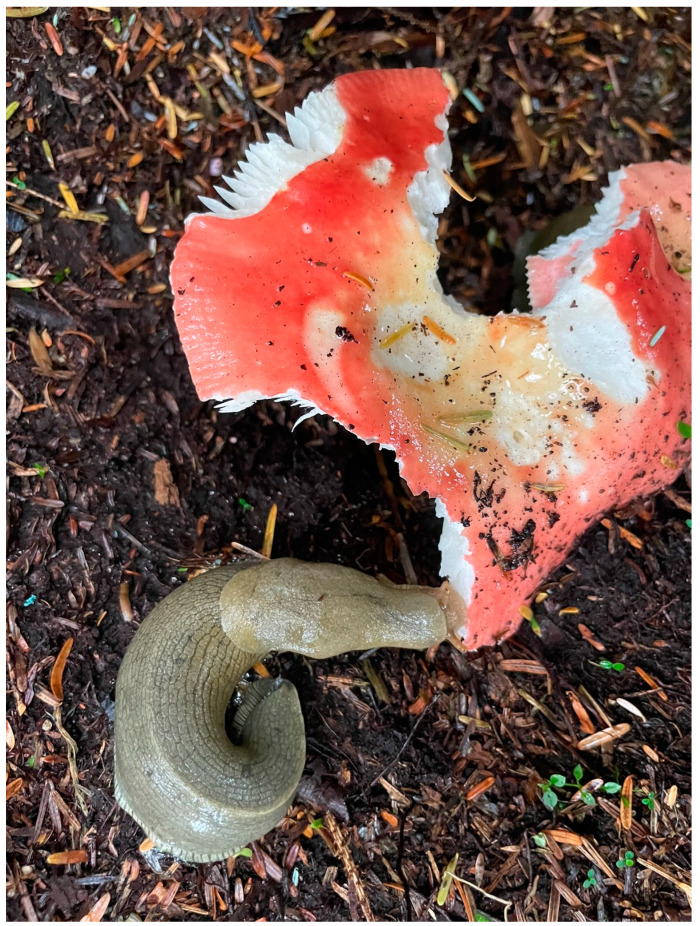
While most mollusks are aquatic there are many terrestrial forms. Pictured here is a Pacific banana slug (*Ariolimax columbianus*), the second largest slug in the world, consuming a wild mushroom in Sitka, Alaska. It is native to the Pacific coast of North America. Providing adequate nutrition to gastropods under human care is critical to providing appropriate welfare for these species. Photograph by Sara Georg.

The gastropods are a class in the phylum Mollusca with over 80,000 freshwater, marine, and terrestrial species. The group includes abalone, conchs, nudibranchs, sea hares, slipper shells, slugs (Figure 3), snails, whelks, and many others.

### 3.2. Natural History

The mollusks (or molluscs) are a diverse phylum of invertebrates that encompass a wide range of species, from snails, clams, and oysters to octopuses, cuttlefish, and squid. They are characterized by a soft body often protected by a hard shell, though not all mollusks have shells. Mollusks exhibit a remarkable variety of forms and adaptations that allow for them to inhabit diverse freshwater, brackish, marine, and terrestrial environments. Mollusks have been a significant part of human history as well, providing sources of food, pearls, shells for tools and ornaments, and even ink (from squid and octopuses) used for writing.

The gastropod’s ventrally flattened foot provides for efficient locomotion along a variety of surfaces. Most gastropods are aquatic with an external shell, muscular foot, respiratory chamber with gills, and a well-developed head rich with sensory organs. Of all the molluscan classes the gastropods contain the most species. Thus, it is hard to make generalizations for the whole group. An exception that makes one easily appreciate this variety is the common garden slug, which lacks a shell, gills, and is terrestrial.. Gastropod feeding and reproductive behaviors are quite varied. Many gastropods are dioecious (display separate sexes), but most terrestrial pulmonates (slugs and snails) are hermaphroditic [14].

**Figure 4:**
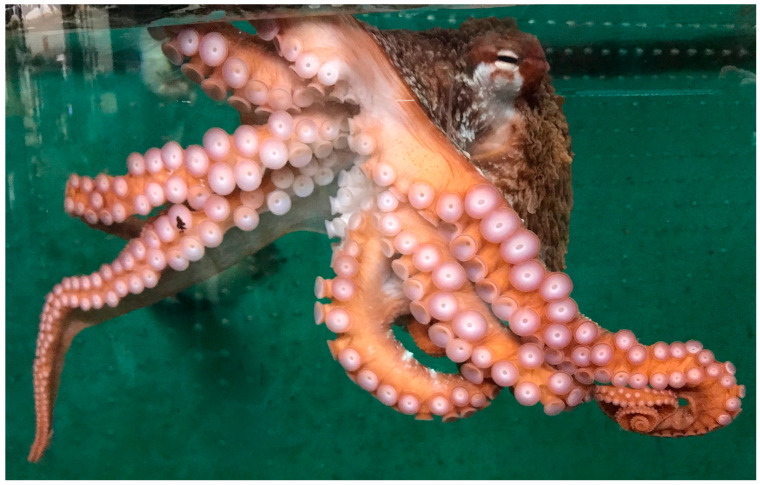
The giant Pacific octopus (*Enteroctopus dofleini*), pictured here, is a very common species displayed in public aquariums and zoos. Although relatively short-lived, these are highly intelligent, sentient animals that must have environmental enrichment and stimulation in order to thrive under human care. Some countries now have laws enforcing certain welfare components for cephalopods. Photograph by GA Lewbart.

There are over 800 species of cephalopods, a group that includes the chambered nautilus, cuttlefish, octopuses (Figure 4), and squids [42]. Detailed scientific accounts have only been published on about 60 of these species [43]. This is a very important group as they serve as food for humans and other animals, have been employed in a variety of important research projects, and are popular aquarium and display animals [43]. Their closed circulatory system, excellent dexterity, acute vision, and intelligence make them fascinating animals to study and observe. Most species are short-lived in the wild and under human care (the chambered nautilus, which can live 20 years, is an exception).

### 3.3. Environment

Mollusks exhibit a wide range of ecological roles, serving as filter feeders, carnivores, herbivores, and scavengers. They play important roles in various ecosystems as both prey and predators.

### 3.4. Nutrition

While most gastropods are carnivorous, especially marine forms [44], there are herbivores, omnivores, parasites, and even a group that displays kleptoplasty, where plants are consumed and chloroplasts retained within the gastropod’s tissues, providing the slug with photosynthetic nourishment [45]. Virtually all species of cephalopods are predatory carnivores and display a number of adaptations and behaviors for this lifestyle [46].

### 3.5. Physical Health

Common problems under human care include anorexia, microbial infections, trauma, and water quality problems. For a detailed summary of cephalopod health and disease problems see Scimeca et al. [47]. Anesthetic, analgesic, and surgical protocols have been established for some species. In Great Britain, an IACUC (Institutional Animal Care and Use Committee) equivalent protocol is required for cephalopod research.

### 3.6. Behavior

Some, like octopuses and squid, are known for their camouflage skills, complex behaviors, and problem-solving abilities. A review paper [48] summarizes gastropod memory and learning while reviewing over a century of important literature. Since gastropod organ systems are relatively simple when compared to vertebrates they are excellent models for studying learning and behavior. One good example is the sea hare *Aplysia californica*), who’s brain has just 20,000 neurons compared to the mammalian brain that may have a trillion neurons. Another advantage of working with *Aplysia* is that its brain cells are very large and can be individually worked on and mapped. Eric Kandel received a Nobel Prize in Physiology or Medicine in 2000 for his work on non-associative learning in *Aplysia* [49].

Cephalopod behavior is an active area of research. Between 2006 and 2015 there were approximately 500 papers published on cephalopod behavior, far exceeding other areas of research including welfare, climate change, cognition, genetics, and neuroscience [43,50]. In recent years, there has been an increase in genomics research [51]. No animal, vertebrate, or invertebrate has received more attention in the area of behavior and neurobiology than the octopus.

Husbandry research on cephalopods is also receiving much attention. Cephalopods, and especially octopi, should be provided with environmental enrichment. Octopi use chemical cues more often than visual cues in selecting prey [52]. Environmental enrichment studies like this, where food items are placed in opaque jars, allow for the animal to utilize chemical cues to find prey.

A variety of complex reproductive behaviors are displayed by cephalopods [13]. Nearly all cephalopods are sexually dimorphic and polygamy is the norm. Inter-male aggression and female selection of males occurs. These reproductive traits and behaviors make cephalopods good models to study vertebrate sexuality [13].

### 3.7. Mental/Psychological Health and Welfare

The more we learn about certain groups of mollusks, especially gastropods and cephalopods, the more we realize these animals are sentient, intelligent creatures, that benefit from environmental enrichment and are negatively affected by inadequate or stressful life support systems and enclosures. Mollusks, especially cephalopods, gastropods, and bivalves, are exploited as food, for public display, the pet trade, and research. The most sentient of these groups, the cephalopods [53,54] and gastropods, are receiving much attention in both the scientific and lay literature, with the former being highly visible in the public’s consciousness, at least in the more developed countries, with movies like *My Octopus Teacher* [55] and books like the *Soul of an Octopus* [56]. The authors of this review are fully invested in the welfare needs of these species and support all efforts to address shortcomings in the handling, care, maintenance, and slaughter of these animals. A recent paper [57] surveyed the 52 public aquariums and zoos on husbandry and veterinary care practices dealing specifically with the giant Pacific octopus (*Enterotopus dofleini*), with a focus on anesthesia and euthanasia. The results of this study are very interesting and indicate that while there is some consistency among institutions, more research, and new information, is required.

## 4. Crustaceans

### 4.1. Taxonomy

The Crustacea are a subphylum in the phylum Arthropoda and are considered a sister group to the Insecta. The most dominant class within the Crustacea are the Malacostraca. Nearly all familiar crustaceans, such as crayfish, lobsters, shrimp, and crabs belong to this class. These crustaceans have a well-developed carapace (the hard shell covering the cephalothorax) and include most of the larger and commercially valuable species.

Within the class Malacostraca, crustaceans are further divided into a number of orders that include:

- Decapoda: crabs, lobsters, shrimp, prawns, hermit crabs, and crayfish.
- Isopoda: organisms like pill pugs (rolly pollies) and woodlice.
- Amphipoda: beach fleas and sand hoppers.
- Euphausiacea: mostly krill, which are small shrimp-like organisms that are essential to many marine food chains.
- Stomatopoda: contains the mantis shrimp that are known for their powerful, sharp claws.

### 4.2. Natural History

The crustaceans are an extremely successful class in the phylum Arthropoda. There are between 35,000 and 55,000 species of crustaceans [14]. Pechenik [58] presents the number as 42,000, and this number has surely increased in the past decade plus. Crustaceans differ from insects and other arthropod classes by having five pairs of cephalic appendages, including two pair of antennae, and a larval stage termed the nauplius that possesses a lone medial eye and three body segments [58]. The arthropods include the well-known shrimp, lobsters, crayfish, crabs, hermit crabs, and barnacles. There are a number of less obvious but still quite numerous taxa including amphipods, brine shrimp, copepods, and isopods among others. In terms of economics this is one of the most important invertebrate groups. Large numbers of crustaceans are used in research, and many are maintained as display animals. Their diverse roles in many aquatic food webs make them essential ecosystem components, influencing nutrient flow and energy. Crustaceans also play a crucial role in aquaculture and fisheries due to their economic value as a food source for humans and domestic animals.

A rich literature of published information supports the fact that decapod crustaceans have a nervous system analogous to the autonomic nervous system of vertebrates [59]. The cardiac ganglion and the stomatogastric ganglion are the two primary ganglia involved in this crustacean autonomic system [59]. Nerve nets, to which these ganglia are connected, innervate all of the major organs. The reader is encouraged to read Shuranova et al., [59] for a detailed review of crustacean neurology. Anyone closely examining a decapod crustacean can quickly tell that they are well equipped with elaborate sense organs in the form of eyes, sensory mouth parts, and antennae [58]. Crustaceans are able to hear, or at least sense vibrations [60]. This ability can likely alter their behavior and movements.

### 4.3. Environment

**Figure 5:**
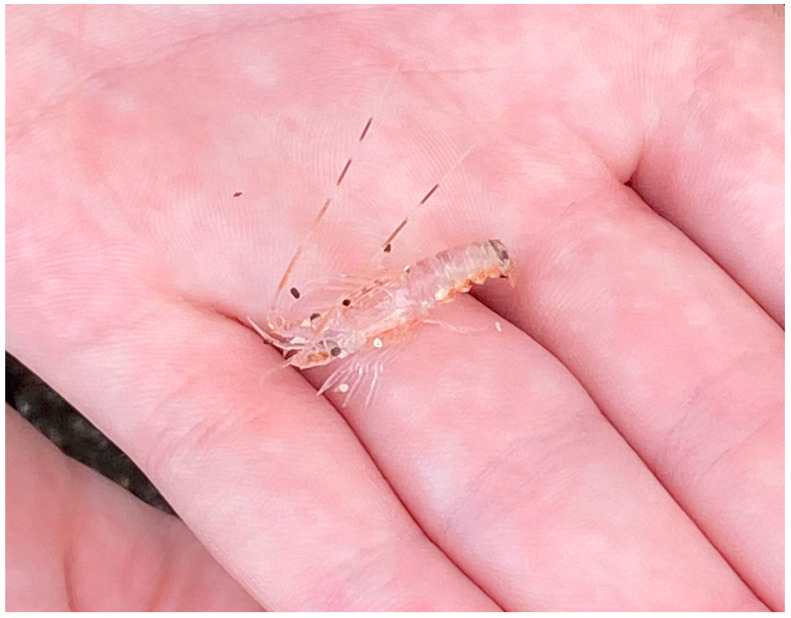
This juvenile spiny lobster (*Panulirus* sp.) from the Galápagos island of Floreana was found on an open beach, vulnerable to predators. Crustaceans, and in particular decapod crustaceans like crabs and lobsters, deserve strong welfare consideration in all capacities where humans are interacting with them. In some countries crustaceans are now protected by laws that address their welfare needs. Photograph by GA Lewbart.

While the majority of species are marine, there are brackish, freshwater, and terrestrial members (although these forms possess an aquatic larval stage). When crustaceans are managed under human care it is critical that they be provided with adequate water quality, nutrition, and appropriate areas of refuge (hiding places). Juvenile decapod crustaceans like crabs and lobsters (Figure 5), while quite numerous compared to the adult forms, are very cryptic to avoid predators.

### 4.4. Nutrition

Crustacean feeding strategies vary widely and are influenced by factors such as life stage, size, and habitat. Crustaceans have diverse diets and feeding strategies that include herbivory, carnivory, detrivory (consuming detritus), scavenging, and filter feeding.

### 4.5. Physical Health

There is a vast literature (entire journals and textbooks) on disease and health problems of crustaceans, focused primarily on the most economically import species, nearly all of which are decapods. For a review of this topic see Hancock-Ronemus et al. [61].

### 4.6. Behavior

Decapod crustaceans are known for their cannibalistic behavior, especially the crabs and lobsters, making these species problematic for aquaculture [62].

Most crustacean behavior research has focused on the order Decapoda decapod in the class Malacostraca that contains over 14,500 species [63]. This large and prominent group includes the shrimp, lobsters, crabs, hermit crabs, crayfish, and prawns.

Much of the behavior research has been centered on aggression as these behaviors are easily observed and crustaceans make an attractive model for vertebrate aggression [64]. The escape response is another area of behavioral research, which in crayfish (*Procambarus clarkii*) involves a series of rapid, giant afferent and efferent neuron-controlled tail flips, which bypass the central nervous system [65,66].

Scientists have tested the effects of isolation on memory and aggressive behavior in decapods. When a *Cherax destructor* (a crayfish) is isolated for 1 month, and then presented to a non-isolated conspecific, the isolated individual usually is the loser in a territorial battle [64]. The findings are just the opposite when the hermit crab *Pagurus samuelis* is isolated for 30 days (the isolated animal is usually the victor) [67].

*Pagurus bernhardus*, a species of hermit crab, has been an important model for studying invertebrate behavior [68,69,70].

A 2020 study determined that a crayfish (*Procambarus clarkii*) exhibits the satiety sequence (BSS) behavior after feeding, similar to many vertebrates [71]. The BSS usually includes grooming, resting, and exploration; grooming and resting in pigeons and rodents [72,73]. Crayfish exhibit all of these behaviors after eating plus something not found in the vertebrate subjects, leg waving [71]. Crayfish (and other crustaceans) are not alone in exhibiting behavioral satiety among invertebrate animals. A 2008 study found that the nematode *Caenorhabditis elegans* can also be a model for satiety in vertebrates [74].

### 4.7. Mental/Psychological Health and Welfare

In recent years, more attention is being paid to crustacean welfare than any other invertebrate group, excluding the cephalopods. Diggles [9] provides a thorough review of crustacean welfare. Crustacean Compassion is an organization based in the United Kingdom focused entirely on crustacean welfare [75] that lobbies hard for current practices reform when it comes to crustacean welfare.

Despite the rich crustacean behavior literature, very few papers focus on animal personality [76], since animal personality had only been studied in a handful of decapods as of 2012 [76].

Perhaps more than any other invertebrate group besides the cephalopods, and perhaps some arachnids, crustaceans deserve our strong consideration when it comes to animal welfare. Many of these species are long-lived, sentient, and interactive creatures that can survive many years or even decades under human care. In these situations, environmental enrichment needs to be considered. In addition, many decapod species are widely consumed by humans, and humane slaughter is a topic that has gained significant traction in recent years.

## 5. Echinoderms

### 5.1. Taxonomy

The echinoderms, like vertebrates, are deuterostomes, meaning the first embryonic opening becomes the anus. They are a phylum of exclusively marine animals, with over 6500 species representing five classes.

**Figure 6:**
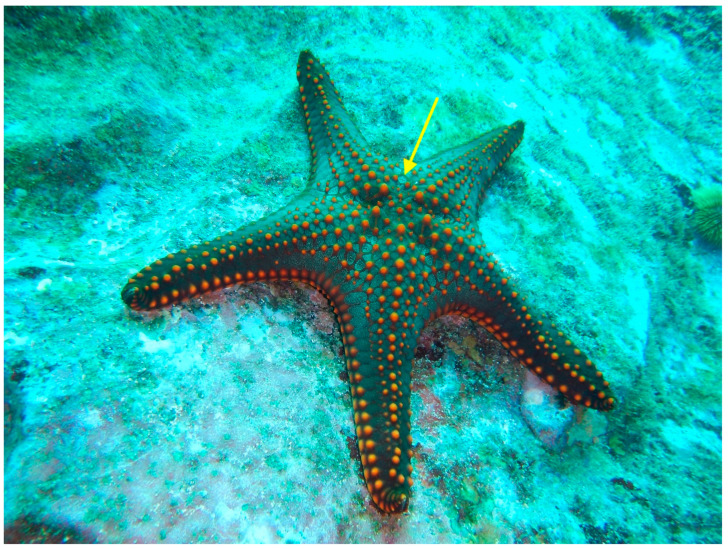
This healthy sea star (*Pentaceraster* sp.) was photographed in the Galápagos Islands in about 10 m of water. The yellow arrow points to the madreporite, or sieve plate, crucial to the water vascular system that all echinoderms depend on. Echinoderms are evolutionarily advanced animals that are developmentally close to vertebrates. In both the wild and under human care they respond to various stimuli and deserve our consideration when being handled, captured, maintained, and slaughtered. Photograph by GA Lewbart.

Asteroidea: this class includes sea stars or starfish. Sea stars have multiple arms radiating from a central disc (Figure 6).

Ophiuroidea: this class includes brittle stars, or ophiuroids, that have slender, distally tapered arms, distinct from the central disc. These arms are usually more flexible than those of sea stars.

Echinoidea: this class includes sand dollars, sea urchins, and sea biscuits. They are characterized by a spherical or flattened body covered in spines.

Crinoidea: this class includes feather stars and sea lilies. Also known as crinoids, they are typically sessile and filter feed with their feathery arms.

Holothuroidea: this class is made up of sea cucumbers. Also known as holothurians, they have elongated, frequently robust, cucumber-like bodies and a thick flexible tunic.

### 5.2. Natural History

All echinoderms possess pentamerous radial symmetry, a water vascular system, and mesodermal calcareous ossicles that comprise the endoskeleton [58]. The water vascular system is used for feeding, respiration, and locomotion. Contractions of the numerous tube feet allow for echinoderms to move and capture prey. Their unique endoskeleton is composed of calcium carbonate ossicles, or plates, embedded in their body wall. These ossicles provide a rigid structure and may form spines or other protective/defensive features.

Echinoderm members include the sea stars (Figure 5), feather stars, brittle stars, sea cucumbers, sea biscuits, and sand dollars. Echinoderms do not have distinct ganglia or a brain but three distinct neural networks [58]. The circumoral motor hyponeural network is most developed in brittle stars (ophuroids). The ectoneural system (also circumoral) receives impulses from the echinoderm’s body surface. Feather stars’ (crinoids) aboral entoneural system is either very reduced or entirely absent in other echinoderms [58].

Echinoderms possess amazing tissue and organ regenerative abilities. If brittle stars or sea stars lose an arm or part of their body, they can regenerate the lost structure(s). Some sea cucumber species are able to voluntarily eviscerate and then regenerate the lost organs.

### 5.3. Environment

The echinoderm phylum is unusual in that all of its members are marine. They are found in all of the earth’s oceans as well as many bays and tributaries.

### 5.4. Nutrition

Echinoderms employ various feeding strategies. Some, like sea urchins, are herbivorous and graze on algae and other plants. Others, like sea stars, are predators that use their tube feet to force open and feed on bivalve mollusk. Sea stars may also act as scavengers. The filter-feeding crinoids extend their feathery arms to secure and ingest plankton.

### 5.5. Physical Health

There is an expanding body of literature on disease and health problems of echinoderms, focused primarily on the most economically import species (those used in research or as food) or species that are inhabitants of coastal areas where disease problems and mortality are quickly recognized. For a review of this topic see Harms [77].

One area where disease and health problems exist for this group under human care are in the always popular “touch tanks”. Many aquariums and zoos have touch tank exhibits where guests are allowed, and even encouraged, to handle aquatic animals [78,79]. Among invertebrates the echinoderms are probably the most represented taxon. It is important that touch tank animals are given time to rest and recuperate from being handled. Most facilities with touch tanks include this “time out” approach as part of the standard operating procedure.

### 5.6. Behavior

Echinoderms exhibit many interesting behaviors. Covering (securing items like algae, marine debris, and shells with their tube feet) is commonly used by echinoids (sea urchins) as camouflage [80,81,82]. In laboratory experiments, urchins exposed to higher water temperatures were less likely to cover, and if they did, the process took longer [82]. Two other urchin behaviors, sheltering and righting, were examined by Zhang et al. [82]. Although righting reflex intervals did not vary between temperature groups, *Strongylocentrotus intermedius* individuals acclimated to warm water sheltered more than those in normal temperature water [82].

The process known as evisceration is one of the most interesting behaviors exhibited by echinoderms, and by any animal for that matter, vertebrate or invertebrate. Sea cucumbers (holothuroids) are able expel entire internal organs, or parts thereof, when attacked or threatened [58,83]. This phenomenon is not a uniform process and different species (there are about 1200 sea cucumber species) display different levels of evisceration and, subsequently, regeneration. Some species only expel sticky noodle-like projections from the respiratory tree called Cuvierian tubules. These projections regrow within a few weeks [58]. Others expel their entire visceral mass, including the respiratory tree, gonads, and GI tract. These animals, in a remarkable feat, can regenerate all of the lost organs [58]. For more information on echinoderm behavior there is an entire volume devoted to echinoderms and cephalopods [84].

### 5.7. Mental/Psychological Health and Welfare

A recent publication (Mauro et al. [85] found that sea urchins (*Arbacia lixula*) exposed to 3 h of 100–200 kHx per second for 3 h exhibited changes in motility. However, differences between protein concentration and enzymatic activities of collected coelomic fluid were not significant between control and experimental groups.

There may not be much in the literature pertaining to echinoderm mental and psychological health but these animals deserve our best effort to provide them with a safe environment, proper nutrition, and environmental enrichment when under human care.

## 6. Chelicerates

### 6.1. Taxonomy

Chelicerates are a subgroup of arthropods that include such diverse animals as arachnids (spider, scorpions, mites, ticks, etc.), horseshoe crabs, and pycnogonid “sea spiders.” Common physical characteristics include a body consisting of two segments, eight walking legs, and paired chelicerae as the first (i.e., most cranial) appendages. The total number of chelicerate species is about 70,000, with spiders (~48,000 species) comprising the majority of those known [14]. Scorpion species (~1500) are much less numerous, and horseshoe crabs are comprised of just four species. Common species found in zoos and aquariums include the following:

- *Brachypelma smithi*, Mexican redknee tarantula
- *Grammostola rosea*, Chilean rose tarantula
- *Avicularia avicularia*, pinktoe tarantula
- *Theraphosa blondi*, goliath birdeater tarantula
- *Latrodectus mactans*, southern black widow
- *Trichonephila inaurata madagascariensis*, red-legged golden orb weaver
- *Pandinus imperator*, common emperor scorpion
- *Limulus polyphemus*, Atlantic horseshoe crab

### 6.2. Natural History

Spiders and scorpions are found on all continents except Antarctica. Since they are more numerous in species, spiders occupy all terrestrial ecosystems [86] and even some aquatic ones. Scorpions tend to inhabit more xeric or desert areas. All are carnivorous, though exceptions are known [87]. The majority of prey consists of insects, with other invertebrates and even small vertebrates sometimes included [86,88,89,90]. Prey are subdued by a combination of envenomation and physical means, with the exception of the non-venomous spider family Uloboridae [86]. Most species of spider and scorpions are sit-and-wait predators, spending the majority of their time solitarily in retreats (e.g., burrows, webs).

Horseshoe crabs are benthic inhabitants of shallow marine waters. *L. polyphemus* is found along the Atlantic coast of the United States and Mexico, while the other three species occur in coastal waters of Asia from India to the Philippines [91]. All are omnivores that feed on smaller invertebrates, algae, and phytoplankton [14,92].

### 6.3. Environment

**Figure 7:**
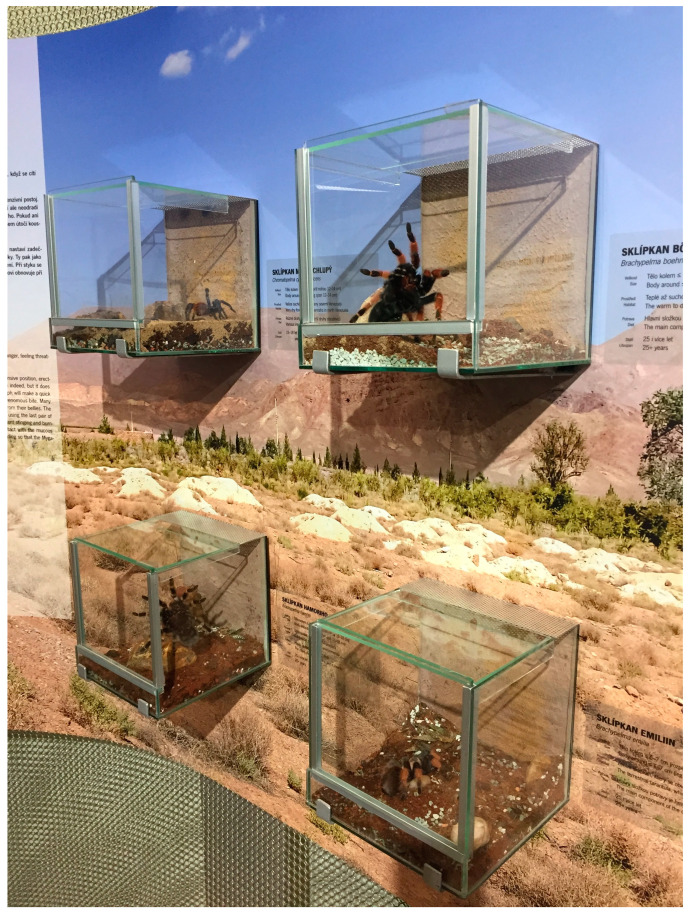
Various species of terrestrial tarantulas in “jewel box” habitats at a zoo. Such displays provide good visibility of the animals. However, several husbandry concerns can be noted: relatively small amount of ground space; small volume of substrate; lack of enrichment materials, especially those from which a retreat could be created; exposure to bright light; poor protection from vibrational stimuli (e.g., guests tapping on habitat walls). It is very unlikely that vertebrates, especially birds or mammals, would be kept in similar enclosures. Photograph by Trevor Zachariah.

Due to their predatory strategy and low activity levels, and out of practicality, spiders and scorpions are often kept in habitats that do not meet even their basic natural history needs, such as the common “jewel boxes” (Figure 7). Scorpions and terrestrial spiders require more ground space, less height, and deeper substrate. Conversely, arboreal and orb-weaving spiders need habitats with greater height and not as much substrate. For many species, natural history information may be scant, but some level of environmental enrichment should be included [93]. This is particularly important for orb-weaving spiders, and the size and form (i.e., two-dimensional v. three-dimensional) of webs should be taken into account [93,94,95]. Enrichment materials are also necessary for spiders and scorpions to make retreats. In addition, the presence or absence of environmental enrichment affects behavior in spiders [48,93].

The design of spider and scorpion habitats can be misguided in that more emphasis is placed on display for guests than welfare of the occupant. Such emphasis can be seen in the use of bright lights, even though most tarantulas and scorpions are nocturnal/crepuscular and are averse to such conditions [96,97]. Also, only minimal glass or plastic barriers may separate guests and habitats. These barriers are often insufficient in shielding animals from vibrational stimuli (e.g., tapping/banging by guests) to which arachnids are sensitive through sensory structures like trichobothria in tarantulas [98] and pectines in scorpions [96]. Stress from repeated vibrational stimuli is likely and can result in negative consequences, such as opisthosomal (abdominal) alopecia from the defensive use of urticating setae of New World tarantulas [97,99].

The parameters for human care of horseshoe crabs as well as concerns for their welfare and survival have been described [5,100,101,102,103,104,105,106,107]. However, in zoos and aquariums, horseshoe crabs are often included in touch pool exhibits, which allow for interaction between guests and their animal inhabitants. The environments of touch pools are often limited compared to larger habitats, including such differences such as shallower/less volume of water, less enrichment, and more contaminants [108]. Additionally, touch pools may have less substrate present, which limits the natural horseshoe crab behaviors of plowing and burying themselves. What effects these differences may have on animal health and behavior are unknown.

### 6.4. Nutrition

While spiders and scorpions are carnivorous, the majority are not specialist feeders, and finding prey items for them is not difficult. However, diets provided in human care are usually limited in variety and complexity, often consisting of just a few species of insects (e.g., house cricket (*Acheta domesticus*). Such monotypic or low-variety diets may predispose spiders and scorpions to nutrient imbalances or deficiencies [109]. The nutrient composition of a number of insect prey species has been documented [110] but, unfortunately, this information is difficult to apply, as nutrient demands of arachnid species are poorly understood [93,97]. It has been shown, however, that prey type and quality can have significant effects on the fitness (i.e., growth, size, survival, reproduction) of predators [93,111,112].

The proper provision of water to tarantulas and scorpions is important. These arachnids depend on open water sources that are large enough and positioned in such a way that their oral openings can contact the water surface [96,97]. Without this, tarantulas and scorpions are prone to dehydration, severe cases of which can affect their ability to move and potentially be fatal [97,113]. The use of cotton balls or sponges in water dishes are not recommended, as they can impede such drinking activity and provide an environment for bacterial growth [96,97]. A small stone or similar item may be added to the water source to prevent prey from drowning and spoiling the water [96,97].

Horseshoe crabs are omnivores, and their dietary habits in the wild have been studied. For *L. polyphemus*, the majority of the diet consists of a wide variety of small invertebrate prey [114]. Despite this finding, most diets in human care consist of a relatively small variety of large invertebrates (e.g., decapods, cephalopods) and teleost fishes [92,115]. This difference in diets may be a factor in the hypoproteinemia frequently found in adult *L. polyphemus*, and possibly in the general poor survival rate of the species in aquaculture [106,115].

### 6.5. Physical Health

As noted previously, spiders and scorpions possess anatomical structures that are sensitive to vibratory stimuli [96,98]. When not properly shielded, habitats may allow for frequent and excessive vibration (e.g., tapping/banging by guests). Also, the use of arachnids as ambassador animals often necessitates putting them in small containers that are easily bumped and jostled. It is difficult to assess how much stress such conditions create for spiders and scorpions. However, a common abnormality of New World tarantulas is opisthosomal alopecia [97]. Many species of New World tarantulas have numerous, minute urticating setae on their caudal body segment that are dispersed by the most caudal pair of legs when the animal is disturbed or stressed [99]. Frequent stress may cause excessive loss of the urticating setae, thus resulting in opisthosomal alopecia.

Dehydration in arachnids can occur due to various causes, with inadequate temperature/humidity and improper water provision common under human care. Mild dehydration may not manifest clinically, but moderate to severe stages can cause significant morbidity and even death [96,97,113]. As an example, spiders have muscles for contraction of their appendages, but rely on hemolymph pressure for extension [86,97]. Moderate to severe dehydration can reduce hemolymph pressure enough that a spider cannot move due to constant contraction of the appendages [97].

Dysecdysis in chelicerates sometimes occurs [96,97,106]. It is unlikely to have one etiology, though animal hydration status may be a prominent consideration in terrestrial species [97]. Many cases are likely multifactorial, involving environmental (e.g., humidity, water quality), nutritional, animal health, and other factors. Optimizing husbandry conditions can prevent most cases.

Trauma is a relatively common occurrence in arachnids [96,97] and occurs in horseshoe crabs [106]. Because of their open circulatory systems, chelicerates are at risk of hemolymph loss and secondary infections whenever the exoskeleton is broken. Hemolymph loss volume does not always correlate with the size of the injury [97]. Animal ambassador programs or touch pools where animals are picked up and handled are opportunities for trauma from dropping or unruly actions of guests. Tarantulas can be easily startled, and when fleeing can sustain severe injuries from falling even short distances. Horseshoe crabs tend to be active when handled and can pose a challenge to proper restraint, especially when turned over for observation of their ventral anatomy.

As mentioned, horseshoe crabs in touch pool habitats may be turned over, often out of the water, as their ventral anatomy is generally difficult to observe routinely. The amount of stress caused by being out of the water is unknown, and indeed, adult animals naturally come out of the water during the annual migratory breeding congregations [14,116,117]. However, physiological changes, such as hypoxia, hypercapnia, acidosis, have been demonstrated in *L. polyphemus* after as little 5 min out of water [118,119]. The long-term effects of such changes from repeated occurrences of handling out of water are unknown.

### 6.6. Behavior

Only little information is available on the effects of human care on chelicerate behavior. One study found significant differences in behavior between wild and laboratory-reared individuals of a cursorial spider [120]. Several studies have found behavioral differences between spiders raised in enriched versus non-enriched environments [121,122,123]. On a practical level, inadequate husbandry can impede natural behaviors. Examples include lack of proper substrate volume that prevents burrowing or burying, or habitat structure that does not allow for normal web building.

Regarding the use of chelicerates as ambassador animals, only one study has examined the effects of direct handling on animal behavior [124]. It was demonstrated that there were significant changes in the behavior and habitat use of *Brachypelma hamorii* tarantulas on days that they were handled compared to days that they were not handled. What effect these changes had on the animal’s welfare was unknown.

### 6.7. Mental/Psychological Health and Welfare

As noted previously, several studies have demonstrated that there are significant differences in behavior of spiders raised in enriched versus non-enriched habitats [121,122,123]. Physical differences in neurologic structures have also been demonstrated in a similar study [125]. What effects these behavioral or anatomical differences may have on animal mental health are unknown. Additionally, the amount of psychological stress that chelicerates experience from inadequate/improper husbandry, handling, and other conditions of human care is also unknown. The authors of this article firmly believe that most chelicerates are sentient beings that deserve not only the best we can offer but strong consideration for improving their welfare through appropriate housing, environmental enrichment, adequate nutrition, and minimal outside stressors.

## 7. Insects

### 7.1. Taxonomy

Insects are the most numerous group of arthropods. Comprised of approximately one million species, they also are three-quarters of the all the world’s animals [12,14,126,127,128]. Common physical characteristics include a body consisting of three segments, six walking legs, compound eyes, a pair of antennae on the head, and wings on the thorax of adults of most species [14]. Beyond this very basic body plan there is enormous variation in all aspects of anatomy. Common types of insects found in zoos and aquariums include the following:

- Orthopterans (grasshoppers, crickets, katydids, etc.)
- Phasmids (walking sticks, leaf insects)
- Mantodeans (mantids)
- Blattarians (cockroaches)
- Coleopterans (beetles)
- Hymenopterans (ants, bees, wasps, etc.)
- Lepidopterans (butterflies, moths)

The great number and variety of insect species make it difficult to go into great detail about the aspects of their welfare in human care.

### 7.2. Natural History

Insects can be found in almost every ecological niche available on land and in freshwater environments, but few in marine environments [14,126]. Their ubiquity relies on many factors, primarily among which are resistance to desiccation and holometabolous development. The former feature is provided by the waterproof cuticle of the exoskeleton and the ability to close the spiracles, or openings, that lead to the tubular tracheae respiratory system. Holometabolous development, also known as complete metamorphosis, is the most common form in insects. It allows for ecological differences in the life stages for resource partitioning and competition avoidance, among other advantages [14].

Obviously, with the huge number of species and variety of insects, it follows that there would be a coincident variety of behaviors, feeding strategies, reproductive strategies, etc. The majority of insects are solitary, but their social arrangements are varied, extending across the spectrum to eusociality in termites, ants, and some bees and wasps.

### 7.3. Environment

In general, insects have a poor tolerance (i.e., rapidly die) for inadequate or improper environmental conditions, so husbandry standards typically need to be precise [129]. Many species are evolved to occupy specific ecological niches and are highly susceptible to changes [126]. However, the detailed needs for most species are not known, necessitating extrapolation from similar/related species and systematic evaluation [126]. This task is made doubly hard when breeding or raising holometabolous insects, as larval stages usually have significantly different needs than adults.

A popular method of displaying insects in zoos is via butterfly houses. To provide entertainment and education, such displays require a variety of butterfly species, each with its own specific husbandry requirements [28]. These requirements (e.g., light, temperature, and humidity cycles) may not always be met adequately, which could lead to stress and possibly shorter lifespans [129]. Additionally, the amount of stress, if any, created by direct interactions between butterflies and guests is unknown.

**Figure 8:**
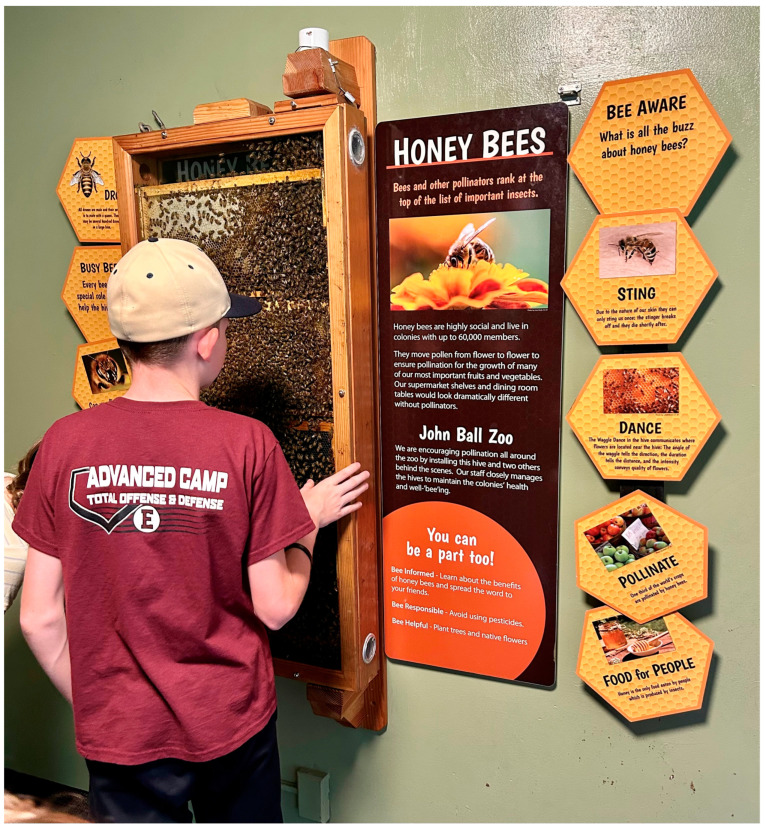
A colony of the eusocial Western honey bee (*Apis mellifera*) on display at a zoo. Honey bee colonies are considered “superorganisms” with welfare concerns focused on the group as a unit, rather than individual bees. The population and organization of a colony gives it buffering capacity against stressors. Important considerations for honey bee colony welfare include appropriate space, temperature, food, and access to fresh water. Photograph by Trevor Zachariah.

Another popular insect display is colonies of the Western honey bee (*Apis mellifera*) (Figure 8). These eusocial animals live in a highly regimented organization with multiple levels, and so the focus of care is on the colony “superorganism”, rather than individual bees [130,131]. The population size and complexity of these superorganisms provides a high capacity for the buffering of stressors, which can make the effects of improper/inadequate husbandry difficult to discern or delayed in expression [131].

### 7.4. Nutrition

As noted previously, insects are incredibly varied in many aspects, and dietary preferences are no exception. Some species or particular life stages are specialists, feeding on one or just a few food sources. Good examples of this are the larvae of monarch (*Danaus plexippus*) and atala (*Eumaeus atala*) butterflies, which exclusively consume milkweed plants (*Asclepias* spp.) and coontie palms (*Zamia integrifolia*), respectively. Other insects are more generalist omnivores, such as cockroaches and the house cricket. Despite the practical knowledge about which species are specialist feeders, the nutritional needs of most insects are not known. Similar to environmental conditions, insect diets should be extrapolated and then systematically evaluated to determine proper quantities and qualities.

### 7.5. Physical Health

In human care, overcrowding and poor hygiene can predispose insects to disease [126,129]. Recommended stocking densities for some species are known, such as the rule of one adult butterfly per square meter [132]. A number of infectious and non-infectious diseases have been described in insects [126], especially those species like honey bees that are of great economic importance [34,131,133,134,135].

Some species of insects, such as the Madagascar hissing cockroach (*Gromphadorhina portentosa*), are popular ambassador animals. Their use in educational settings often involves handling, which puts them at risk of trauma from dropping, excessive pressure, or catching of limbs on clothes [126]. Traumatic breaches of the exoskeleton can cause fatal loss of hemolymph, and even sub-lethal injuries may predispose to other morbidities (e.g., infection, inability to fly due to wing damage/loss).

Handling of insects may also cause physiologic stress, as demonstrated by an increased heart rate in all life stages of the monarch butterfly [136]. It is unknown if the stress from repeated handling leads to deleterious consequences, such as changes to immune responses.

### 7.6. Behavior

Under human care, insects may not be able to perform the full repertoire of their natural behaviors. Examples include butterflies that will not lay eggs without species-specific host plants [129], or the migratory and swarming behavior of locusts. Thus, all efforts should be made to provide care that maximizes a species’ behavioral capacity [50,137]. The start of such a process should likely involve careful consideration of what species are chosen to be kept in human care.

### 7.7. Mental/Psychological Health and Welfare

A growing body of research has shown that across many taxa, insects are capable of varied cognitive capabilities and likely also experience emotional states such as stress [138]. The presence of personalities in insects and other invertebrates, including social species, has also been argued (Mather and Carere, 2019). The psychological consequences of such are that personality differences may affect differences in coping with stress [139]. Stress responses can result in beneficial effects, such as anti-predator behavioral changes [140,141,142]. However, chronic, repeated stress can also have negative behavioral effects, such as decreased diet consumption and weight loss [140].

As with crustaceans and chelicerates, insects deserve our attention as they are sentient beings though most are not as long-lived as the aforementioned groups. Insects are widely consumed in some parts of the world and are frequently exploited as pets, display animals, and for research. Recent work by Barrett and Fischer [143] addresses the important topic of insect farming and welfare. The Insects as Food and Feed (IAFF) currently raises over 1 trillion individuals per year and this number is growing. Most are crickets, black soldier flies, and mealworms [143]. While addressing welfare in these situations can be challenging, the conversation needs to be initiated, and attempts made to keep animal welfare as one of the key parameters of this industry.

## 8. Myriapods

### 8.1. Taxonomy

Myriapods consist of two groups, chilopods (centipedes) and diplopods (millipedes), with approximately 2800 and 10,000 species, respectively [14]. Common physical characteristics include an elongate body of multiple leg-bearing segments and a pair of antennae on the head. Significant anatomical differences exist between the two groups, in that centipedes are dorsoventrally flattened with one pair of legs per segment, while millipedes are cylindrical with two pairs of legs per segment [144]. Another unique feature of centipedes is a pair of venomous fangs, called forcipules, on the first trunk segment. Common species found in zoos and aquariums include the following:

- *Archispirostreptus gigas*, African giant millipede
- *Sechelleptus seychellarum*, Seychelles giant millipede
- *Epibolus pulchripes*, Mombassan train millipede
- *Scolopendra* spp., giant centipedes

### 8.2. Natural History

Myriapods are found in many temperate to tropical areas, with larger species typically found in warmer regions. All are adapted to burrowing and living in leaf litter and other plant-based substrates [144]. Unlike insects, myriapods do not have a waterproof cuticle nor spiracles that can close; thus, they require humid environments or else risk desiccation [140]. Beyond these basics, lifestyles of the two myriapod groups diverge greatly.

Centipedes are solitary, flexible, fast-moving carnivores, preying mostly on insects and other invertebrates, and sometimes small vertebrates [14,144]. Millipedes are slow-moving and mostly herbivorous/detritivorous, which helps nutrient recycling in their environments [144]. Their exoskeletons are heavily calcified and relatively stiff [14,144]. Some species are social, but most are at least tolerant of other individuals and assemblages.

### 8.3. Environment

Similar to terrestrial arachnids, myriapods require a good amount of substrate and enrichment in their habitats. There are several significant reasons for this. One, is that they are cursorial/fossorial, so should be allowed to express those natural behaviors. Two, as stated previously, myriapods are particularly sensitive to desiccation, so the substrate can help trap moisture in the habitat. Lastly, they are photophobic [144], so burrows and retreats prevent stress from bright lights often used for display purposes.

### 8.4. Nutrition

As generalist carnivores, centipedes consume many different types of prey. In human care, the variety of prey items offered should be maximized as much as possible, including vertebrates for larger species. The differing nutrient content of prey species helps to provide a more nutritionally complete diet.

Variety is also important for herbivorous millipedes. However, due to the amount of calcium incorporated into their exoskeletons, calcium supplementation (e.g., cuttlebone) is usually necessary.

Similar to arachnids, all myriapods require a standing source of open water from which to drink [144]. The lack of such a feature in their habitats may predispose them to desiccation.

### 8.5. Physical Health

**Figure 9:**
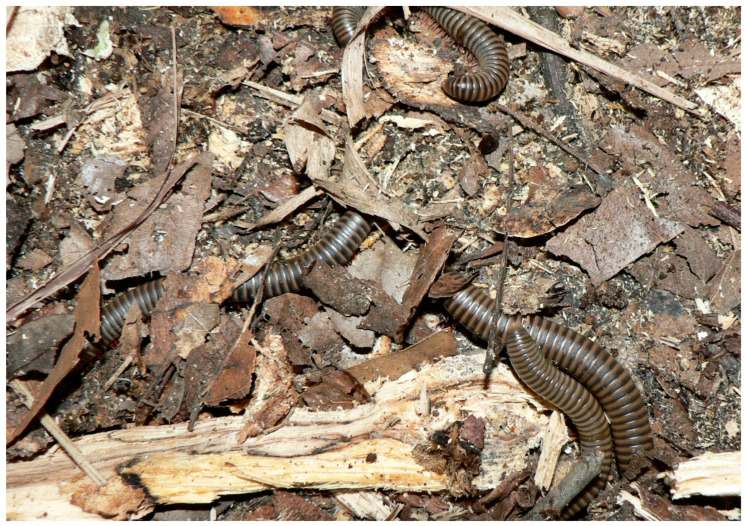
Pictured here are several terrestrial millipedes from the state of Florida. These animals are frequently fossorial, although they can be observed on sidewalks, roadways, and on lawns or in gardens. While some excrete a slightly noxious substance when disturbed, they are relatively harmless to humans, and are fascinating to observe. Some are kept as pets or displayed in zoological collections. When maintained under human care they deserve welfare consideration with regard to habitat, nutrition, enrichment, and safety from predators and other threats. Photograph by D Deresienski.

Millipedes (Figure 9), especially the larger species, are commonly used as ambassador animals. Handling is usually safe, but does introduce the possibility of accidental drops, and trauma to the exoskeleton can lead to life-threatening injuries (i.e., hemolymph loss, secondary infection). Additionally, most millipedes can exude noxious substances (e.g., aldehydes, quinones, phenols, iodine, chlorine, hydrogen cyanide), the composition of which vary by species [14]. Such substances can irritate skin or startle a handler, increasing the chances of an animal being dropped. Due to their venomous forcipules and aggressive nature, centipedes are rarely handled.

Dysecdysis occurs in both centipedes and millipedes but is a more significant concern in the latter [144]. This etiology is likely multifactorial in most cases, with the majority of factors related to improper husbandry (e.g., inadequate humidity). It is also possible that a deficiency of calcium could contribute to dysecdysis in millipedes.

### 8.6. Behavior

There is a dearth of information pertaining to how myriapod behavior is affected by being in human care. At least one study has demonstrated that millipedes possess the basic form of olfactory learning [145]. This suggests that myriapods may experience significant behavioral changes in response to conditions under human care. Natural behaviors can be impeded, such as when an improper volume of substrate prevents burrowing.

### 8.7. Mental/Psychological Health and Welfare

To the authors’ knowledge, no information regarding the mental/psychological health of myriapods is currently available. Unlike most of the taxa highlighted in this article, centipedes and millipedes are minimally consumed by humans, are not widely displayed in zoos (although there are exceptions), are not commonly kept as pets, and do not contain important research models like the mollusks (e.g., *Aplysia*; sea hares and *Loligo*; squid), crustaceans (e.g., mysid shrimp) echinoderms (sea urchins), chelicerates (e.g., *Limulus*; horseshoe crabs), and insects (*Drosophila*; fruit flies). Regardless, they are interactive creatures that can live for several years and deserve all of the welfare consideration and parameters addressed with the more “popular” taxa.

## 9. Conclusions

It goes without saying that since this is an article reviewing the topic of welfare for invertebrates, we, the authors, believe strongly in this topic. More importantly, our goal is to bring attention to this topic, particularly to those people and stakeholders who may be more focused on vertebrate animals. Anyone interacting with or closely observing an octopus, lobster, or tarantula cannot help but notice the level of awareness, mobility in response to stimulation, and employment of the various senses. While in graduate school one of GAL’s professors, a malacologist (someone who studies mollusks), said that if cephalopods had ever evolved to be terrestrial they would rule the world. With the cephalopod’s intelligence, visual acuity, and manual dexterity, I have little doubt of this statement’s validity. While cephalopods may not be ruling the world, they and their millions of invertebrate species counterparts are an important, if not essential, part of our world. As such they deserve our attention, respect, and humanity. The best way for animal health, husbandry, and veterinary professionals to approach invertebrate welfare is to imagine how one might treat these animals if they had a backbone.

## Data Availability

Not applicable.
